# The Unfortunate Case of a Pulmonary Artery Bullet Embolism

**DOI:** 10.7759/cureus.94028

**Published:** 2025-10-07

**Authors:** Jason Stanton, Allyson Daly, Matthew J Billy, Brian Frank, Monty Littlejohn

**Affiliations:** 1 General Surgery, Geisinger Community Medical Center, Scranton, USA; 2 Trauma Surgery, Geisinger Community Medical Center, Scranton, USA

**Keywords:** bullet embolectomy, bullet embolism, bullet migration, retained bullet, ­trauma

## Abstract

Bullet embolism is a rare occurrence, and the relative paucity of literature makes the management of a bullet embolism challenging. There is a lack of information to help guide the treatment of this pathology. This case details a patient who suffered a gunshot wound to the thigh and was subsequently found to have a retained bullet in the left pulmonary arterial vasculature. The patient remained asymptomatic and was managed conservatively without complication. Treatment of venous bullet embolism is not well studied, given the limited examples in the current literature; however, this case suggests that observation alone is appropriate in an asymptomatic patient.

## Introduction

Bullet embolism is a rare event and occurs when a projectile enters a blood vessel and follows the flow of blood to lodge at a distant site [1,2]. Fewer than 300 case reports of bullet embolism have been reported in the literature, with an incidence rate of 0.3-1.1% noted in military populations [3]. The diagnosis can be delayed or missed entirely due to its potentially asymptomatic presentation, especially when venous embolism occurs. The appropriate management of venous bullet embolism remains controversial given the low incidence and often asymptomatic patient, and oftentimes the appropriate management is driven by the clinical presentation.

Bullet emboli can occur either through arterial or venous circulation. The conditions must be just right for a bullet embolism to occur: the physics of the bullet entry as well as amount of energy being just enough to enter the patient without exiting the body, the trajectory must be perfect to enter a vessel, and the size of the bullet or shrapnel must remain small enough to pass through the vascular system while also not lodging at the injury site [1]. Without all of these factors occurring, it is unlikely for an embolization to occur. When they do occur, arterial emboli are typically symptomatic, resulting in acute ischemia, which usually leads to earlier diagnosis [3]. On the other hand, venous bullet emboli are more insidious, with projectiles commonly traveling through the venous system to the right heart following the natural flow of blood, ultimately lodging in the pulmonary arterial vasculature [1,2]. Complications that have been reported include embolism, thrombosis, ischemia to downstream tissue, erosion of the foreign body, infection from retained shrapnel, and even death [4-7]. Clinical presentation ranges from asymptomatic to symptoms mimicking pulmonary embolism when venous in nature, including chest pain, dyspnea, or hypoxia. Even if initially asymptomatic, there is a 20% to 25% risk of complication after venous bullet embolism [3]. Diagnosis typically relies on a combination of clinical index of suspicion, imaging studies, and knowledge of the traumatic mechanism and the number of bullet wounds that exist.

This case illustrates a rare and complex case of venous bullet embolism. An 81-year-old female who sustained a gunshot wound to the right thigh, followed by a motor vehicle accident en route to the hospital, is presented. The finding of a single identifiable entry wound without an exit wound suggested a retained bullet, and subsequent studies revealed that the bullet was within the left pulmonary artery. This case emphasizes the importance of maintaining a high index of suspicion for intravascular migration of ballistic material, especially when there is no identifiable exit wound. The insufficient literature and relatively rare occurrence of this phenomenon create a challenge for the trauma or vascular surgeon when managing these patients and their complications.

## Case presentation

This case presents an 81-year-old female involved in an unfortunate sequence of traumas before her presentation. She initially suffered a gunshot wound to her right thigh after she fell getting out of bed with a holstered firearm. While being transported to the hospital, the ambulance she was in was then struck by another vehicle, further complicating the scenario.

**Figure 1:**
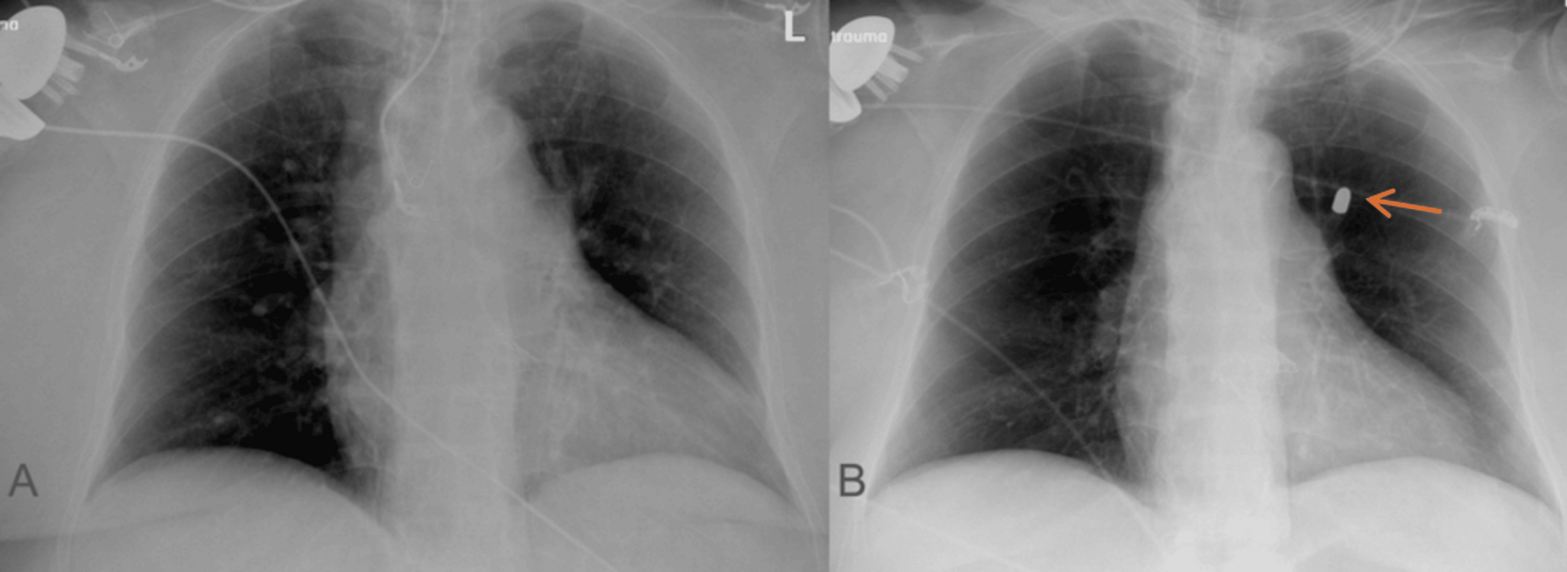
(A) A prior chest X-ray from one month before presentation depicting no evidence of a retained bullet. (B) A chest X-ray on initial presentation with evidence of a retained bullet in the left chest (orange arrow).

**Figure 2:**
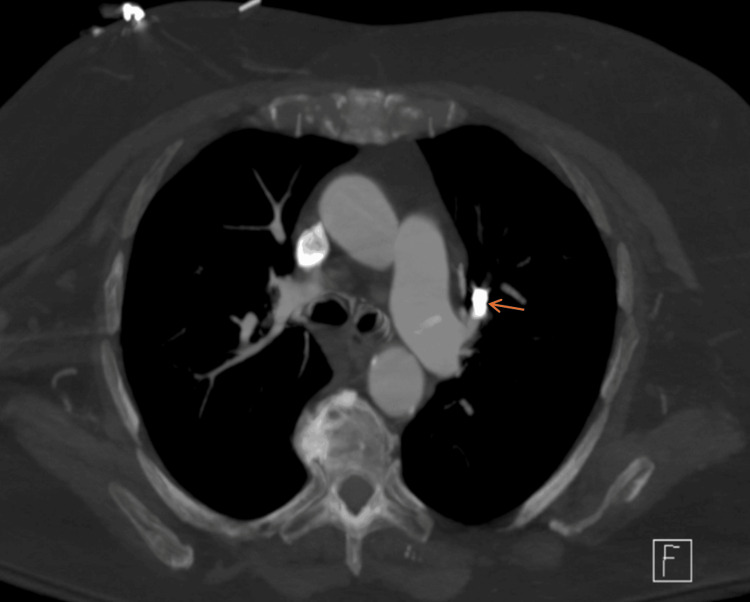
CT scan on the day of presentation showing a metallic foreign body within the left pulmonary artery (orange arrow).

**Figure 3:**
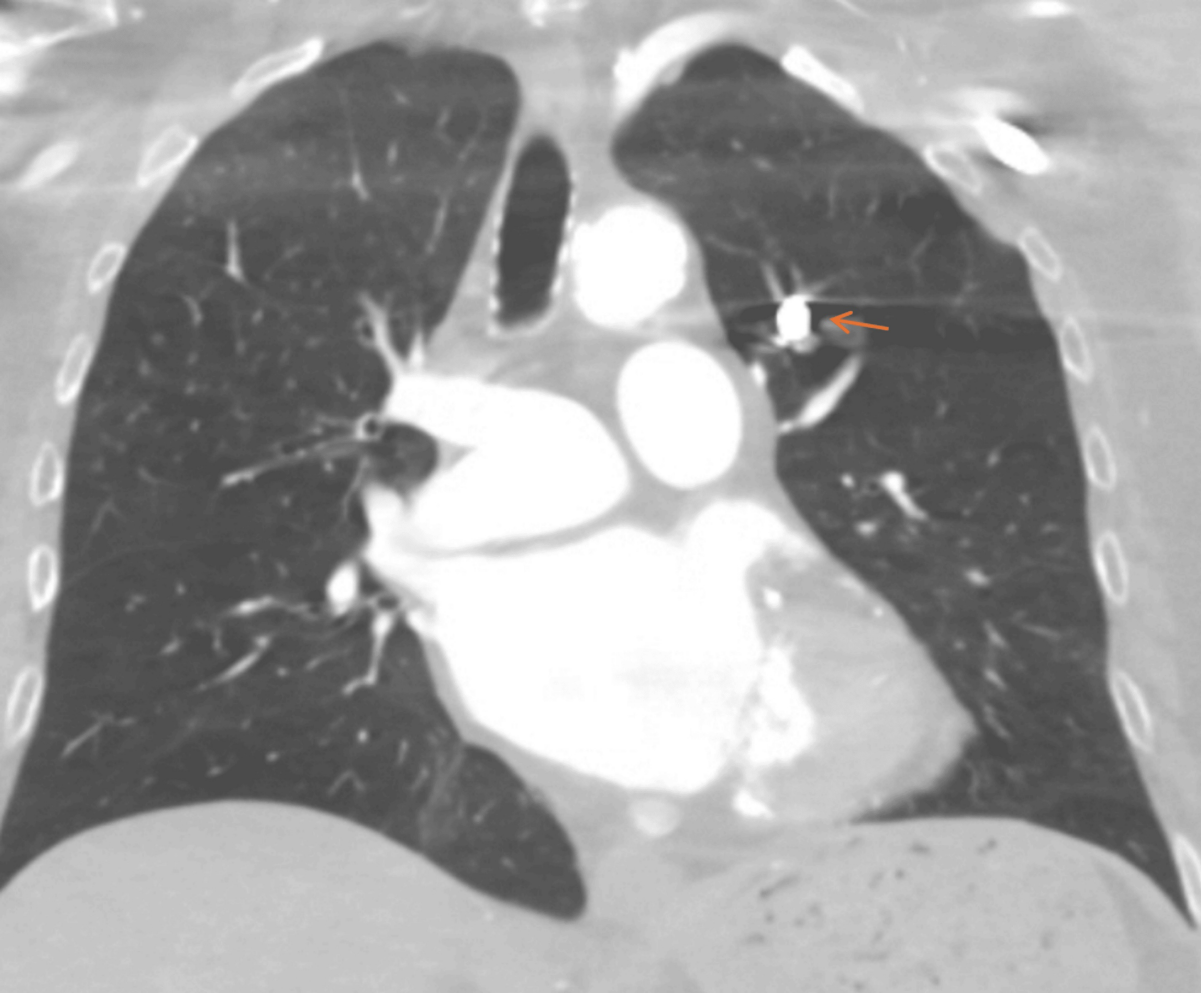
Coronal CT scan showing the same retained metallic object in the left pulmonary artery consistent with a retained bullet (orange arrow).

On arrival, she was alert and hemodynamically stable. Physical examination revealed a single bullet wound to the anteromedial thigh with no exit wound. Given the location, there was concern for vascular compromise, and on further evaluation, there were palpable but diminished pulses in the right lower extremity, raising concern for vascular injury. Initial trauma imaging, including X-rays and CT imaging of the right lower extremity, demonstrated injury to both the right femoral artery and vein, but no retained projectile was visualized in the extremity (Figure 1). Additional CT of the chest as part of the initial trauma evaluation revealed a metallic foreign body consistent with a bullet, lodged in a branch of the left pulmonary artery (Figures 2, 3). There was no evidence of other intrathoracic pathology, including pneumothorax, pulmonary contusion, or associated rib fractures, to suggest chest trauma. Further review of imaging confirmed that no bullet was retained in the right thigh, and the patient denied any history of prior gunshot wounds.

**Figure 4:**
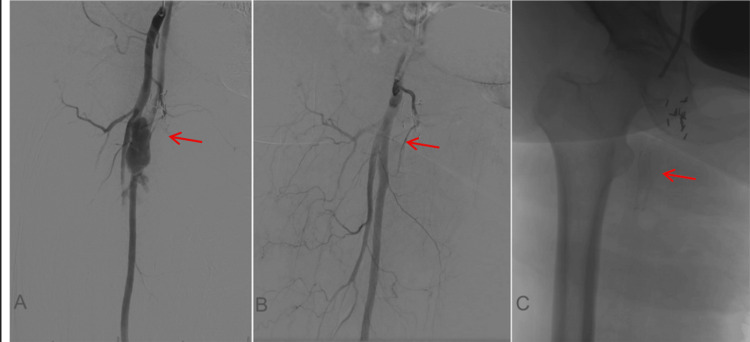
(A) Angiography on the day of presentation showing active extravasation of the left femoral artery (red arrow). (B and C) Successfully stented left femoral artery (red arrow).

Interventional Radiology was ultimately consulted, given the finding of extravasation of blood from the femoral artery, and the patient underwent successful endovascular stenting (Figure 4). The patient did not require surgical exploration at the time due to the stable hemodynamics and a successful endovascular repair.

Given the clinical setting and imaging findings, a diagnosis of venous bullet embolism was made from the femoral vein to the pulmonary artery. The patient remained asymptomatic from a pulmonary standpoint without the need for supplemental oxygenation. After a multidisciplinary discussion between trauma surgery, interventional radiology, and cardiothoracic surgery, it was decided to manage this conservatively. Conservative management was ultimately decided as this patient remained asymptomatic with a segmental pulmonary artery location, bullet diameter less than 5 mm, and hemodynamic stability. She ultimately remained stable throughout the rest of her hospital course and was discharged uneventfully without pulmonary complications. She was seen in follow up where she remained asymptomatic with repeat imaging suggesting a stable location of the bullet embolism.

## Discussion

Bullet embolism is a rare but potentially serious complication of penetrating trauma. Venous bullet embolism, as was presented in this case, accounts for fewer than 25% of reported cases and often presents a diagnostic challenge due to its subtle clinical manifestations [7]. When it does occur, most commonly, the bullet will embolize to the pulmonary vasculature, following the flow of blood as it returns to the right heart and enters the pulmonary circulation. A perfect combination of conditions must be met for bullet venous embolization to occur. The projectile must have the trajectory to penetrate the vein with just enough energy to not exit the body, remain small enough to traverse the venous system, and not become lodged in the surrounding tissue [1]. In this patient, the bullet likely entered the femoral vein and followed the flow of blood through the inferior vena cava to the right atrium and ventricle, ultimately becoming stuck in a branch of the left pulmonary artery.

This case highlights the importance of thorough imaging in trauma evaluation, especially in the setting of multi-mechanistic trauma. This patient had an initial chest X-ray, which showed a metallic foreign body that was later confirmed to be a bullet after there was no identifiable exit wound or other imaging to suggest a retained bullet at the site of the injury in the thigh. This finding emphasizes the need for a high clinical index of suspicion when other imaging findings are inconsistent with clinical examination or history. Furthermore, this case exemplifies the importance of identifying the appropriate trajectory of penetrating trauma as well as ensuring an even number of the sum of bullets and entry/exit wounds [7]. When this number is not even, additional workup should identify the retained bullet, and bullet embolism should be suspected if it is outside the predicted trajectory. Additionally, knowledge of blood flow is important when attempting to establish the pathway a bullet may have taken if it enters the bloodstream.

Interestingly, this patient remained entirely asymptomatic from a pulmonary standpoint. This is consistent with other venous bullet embolism cases that have been reported. Symptomatic presentations can be similar to what would be seen with pulmonary embolism, including dyspnea, chest pain, or hemoptysis [7]. However, in the setting of bullet venous embolism, this case suggests that the asymptomatic patient can be managed conservatively. This is consistent with recent literature that demonstrates successful conservative management in 70% of pulmonary artery emboli cases, though 25% of initially asymptomatic patients may develop delayed complications, making careful selection criteria and long-term follow-up essential [8]. The approach to appropriate management of such a rare pathology ultimately depends on the clinical presentation, location of the embolus, bullet size, risk of complications, and patient comorbidities. Surgical exploration or endovascular retrieval may be indicated in symptomatic cases, central emboli, or bullets at risk of migration and/or erosion [9-11]. In this case, conservative management was appropriate given the distal location of the embolus and absence of symptoms.

## Conclusions

Bullet embolism is an uncommon and unusual complication of penetrating trauma, particularly in the venous system. This case presents a bullet migrating from the femoral vein to the pulmonary artery in an otherwise asymptomatic elderly patient with successful conservative management. This patient was seen in follow-up and remained clinically stable, suggesting the success of conservative management, with repeat imaging showing the stable location of the bullet embolism. Clinicians should maintain a high clinical index of suspicion in trauma patients with unexplained imaging findings, when a bullet is not recovered at the expected site, or there is a discrepancy in the number of bullet wounds and the number of bullets on further imaging. Early recognition, multidisciplinary discussion, and individualized management are key to optimizing outcomes in these unusual but potentially devastating cases. Additionally, this case suggests that conservative management can be performed in an asymptomatic patient with a more distal pulmonary artery location, bullet diameter less than 5 mm, and without evidence of malperfusion to distal tissue.
